# The *Bacillus anthracis *chromosome contains four conserved, excision-proficient, putative prophages

**DOI:** 10.1186/1471-2180-6-34

**Published:** 2006-04-06

**Authors:** Shanmuga Sozhamannan, Michael D Chute, Farrell D McAfee, Derrick E Fouts, Arya Akmal, Darrell R Galloway, Alfred Mateczun, Leslie W Baillie, Timothy D Read

**Affiliations:** 1 Biological Defense Research Directorate, Naval Medical Research Center, 503 Robert Grant Avenue, Silver Spring, Maryland 20852, USA; 2 The Institute for Genomic Research, Rockville, Maryland 20850, USA; 3 University of Maryland Medical Biotechnology Center, Baltimore, Maryland 21201, USA

## Abstract

**Background:**

*Bacillus anthracis *is considered to be a recently emerged clone within the *Bacillus cereus sensu lato *group. The *B. anthracis *genome sequence contains four putative lambdoid prophages. We undertook this study in order to understand whether the four prophages are unique to *B. anthracis *and whether they produce active phages.

**Results:**

More than 300 geographically and temporally divergent isolates of *B. anthracis *and its near neighbors were screened by PCR for the presence of specific DNA sequences from each prophage region. Every isolate of *B. anthracis *screened by PCR was found to produce all four phage-specific amplicons whereas none of the non-*B. anthracis *isolates, produced more than one phage-specific amplicon. Excision of prophages could be detected by a PCR based assay for *attP *sites on extra-chromosomal phage circles and for *attB *sites on phage-excised chromosomes. SYBR-green real-time PCR assays indicated that prophage excision occurs at very low frequencies (2 × 10^-5 ^- 8 × 10^-8^/cell). Induction with mitomycin C increased the frequency of excision of one of the prophages by approximately 250 fold. All four prophages appear to be defective since, mitomycin C induced culture did not release any viable phage particle or lyse the cells or reveal any phage particle under electron microscopic examination.

**Conclusion:**

The retention of all four putative prophage regions across all tested strains of *B. anthracis *is further evidence of the very recent emergence of this lineage and the prophage regions may be useful for differentiating the *B. anthracis *chromosome from that of its neighbors. All four prophages can excise at low frequencies, but are apparently defective in phage production.

## Background

The genome sequence of the bioterrorism pathogen, *Bacillus anthracis *Ames strain, revealed the presence of four putative lambdoid prophages (designated lambdaBa0l, lambdaBa02, lambdaBa03 and lambdaBa04), constituting about 3% of the 5.2 Mbase main chromosome [1]. Comparative genomic hybridization studies showed that the prophage regions made up a higher percentage of the unique genes in *B. anthracis *not found in 19 other *Bacillus cereus *group strains [1]. DNA sequences of all completed and shotgun genome sequences of the diverse *B. anthracis *strains submitted to genbank (Genbank IDs: AE016879, AE017334, AAAC01000001, AAEQ00000000, AAER00000000,. AAES00000000, AAEN00000000, AAE00000000, AAEP00000000) also contain these prophage sequences with >99% nucleotide sequence identity. *B. anthracis *appears to be a recently emerged clone within the *B. cereus sensu lato *group [2-6]. Multilocus sequence typing (MLST) and other strain typing studies have revealed extensive similarities and very few differences among widely distributed strains [1,3-5,7-12]. While other *B. cereus *group genomes also contain lambdoid prophages, these generally contain genes with little DNA sequence homology to *B. anthracis *prophage genes [1,13-16]. Furthermore, analysis of the four other sequenced *B. cereus *genomes (Genbank accession numbers ATCC 10987, AE017194; ATCC 14579, AE016877; G9241, AAEK00000000; ZK, CP000001) revealed that prophages are generally inserted at different chromosomal loci than *B. anthracis*, except in *B. cereus *ZK strain that contains an element very similar to *B. anthracis *lambdaBa01 inserted at the same locus. Interestingly, at the same locus in the *B. cereus *14579 chromosome, is a defective prophage, phi6A53, with little similarity to *B. anthracis *lambdaBa0l. These findings probably reflect what has been observed in other bacterial species: prophage acquisition and loss is quite dynamic, and the gene pool of phages that infect the *B. cereus *group is larger and more diverse than that of the rest of the chromosome [1,13-16].

Prophages, like plasmids, conjugative transposons, insertion sequences, introns and other elements, make up a mobile portion of bacterial genome subject to frequent horizontal exchange that often account for large-scale genomic rearrangements and insertions and deletions in bacterial chromosomes. These mobile elements often encode traits such as virulence markers and antibiotic resistance determinants, which confer selective advantages for the host bacterium in various environments [17-19].

The role of prophages in the pathogenic life cycle of *B. anthracis *is not known and the majority of the genes on the *B. anthracis *prophages do not have an assigned function. In our attempts to initiate a study of the role of the prophages, it was determined that markers for the four prophages are conserved in diverse *B. anthracis *strains and shown that all four prophages are able to excise from the genome but fail to form viable phage particles and hence appear to be defective.

## Results

### *B. anthracis *prophages are common to all strains

Earlier comparative genome sequence analysis of *B. anthracis *and 19 of its close neighbors indicated the absence of the four *B. anthracis *prophages in other strains [1]. The presence of these or similar prophages in a larger collection of *Bacillus *spp strains was tested to gain insights into the evolutionary history of *B. anthracis *as a species. The PCR primers used in the screening were designed to amplify genes that were found to be unique to each *B. anthracis *prophage from comparative genomic hybridization studies [16]. The PCR primers and the expected sizes of the PCR products are listed in Table 1. A set of 300 strains was screened by PCR. It included 192 *B. anthracis *strains from the Biological Defense Research Directorate (BDRD) collection isolated from different geographic regions around the world over a time span of several decades. The set also included 24 diverse genotypic strains identified by the multi-locus variable number of tandem repeat methodology [4]. The set of non-*B. anthracis *strains was primarily from the collection of F. Priest [12] and included 60 *B. cereus *strains and 48 other *Bacilli *spp strains. These strains have been characterized extensively by MLST and grouped into distinct sequence types [12]. All *B. anthracis *strain DNAs produced all the four phage-specific amplicons.

Out of the 108 non-*anthracis *strains, 88 strains did not amplify any of *the B. anthracis *phage specific amplicons whereas 20 of them amplified only lambdaBa02 specific DNA fragment. Thus, presence of all the four prophages appears to be a unique feature of *B. anthracis *strains.

**Figure 1:**
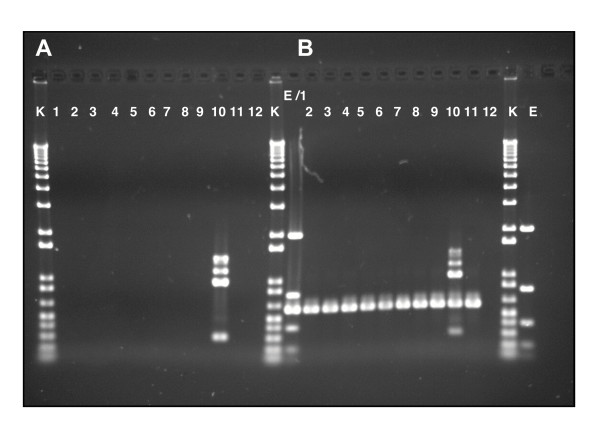
**Multiplex PCR assay for confirmation of *B. anthracis *DNA**. Multiplex PCR analysis of *B. cereus *and *B. anthracis *strains using primers (ORF01192 F-R, ORF02190 F-R, ORF03655 F-R and lambda Ba02 #7 For-Rev). K-1 kb plus ladder (Invitrogen, Inc); E-E-gel low-range marker (Invitrogen, Inc). Lanes 1–9 are *B. cereus *strains; S74, S363, SPS2, F3080B, F3942/87, F4801/72, m1292, F4801 and S710 respectively. Lane 10-*B. anthracis *strain 34F2, Lane 11-*B. cereus *G9241 and Lane 12-reaction control. Panel A reactions were carried out with the multiplex primer set and panel B reactions were carried out with a primer pair for a house-keeping gene *gmk *in addition to the multiplex primer set.

Further verification of the presence of all four phage specific sequences was done by a multiplex PCR assay. The primers used for this assay and the expected sizes of the amplification products are listed in Table 1. All the 192 *B. anthracis *strains but none of the 108 non-*anthracis *strains produced four amplicons of the expected lengths. Inclusion of a pair of primers targeted to a housekeeping gene (*gmk*) [12] as a positive control in the multiplex reaction resulted in amplification of that fragment in all strains tested. The results with a fraction of the strains tested are shown in Figure 1a and 1b. As expected from simplex PCR results described above, all the *B. anthracis *strain DNAs produced all the four PCR products in the multiplex assay. These data indicated that the sequences of the prophage regions might be useful for differentiating the *B. anthracis *chromosome from that of its neighbors.

### All four *Bacillus anthracis *prophages are excision-proficient

**Figure 2:**
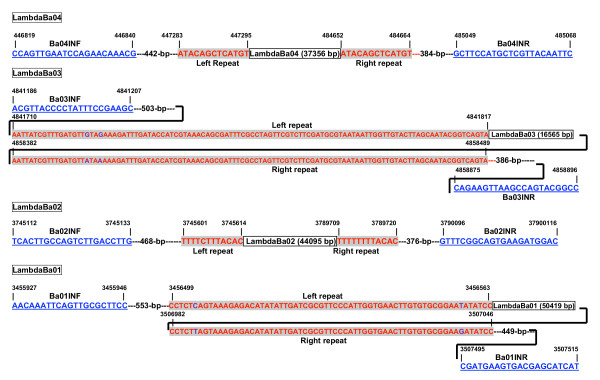
**DNA sequences around the prophage insertion sites in *B. anthracis *chromosome**. The genomic positions in the strain Ames ancestor (Genbank accession number: AE017334) are indicated above the sequence. The sequences of the primers used in PCR of prophage excised chromosomal *attB *sites are underlined. The putative *att *sites, referred as left and right repeats, flanking the prophage genome (indicated by boxes with the phage genome size in bp), are highlighted. The left and right repeats of prophages lambda Ba01 and Ba03 are not perfect and the 2 mismatched bases are indicated in different color.

The stability of *B. anthracis *prophages was analyzed by determining their excision proficiency. The *B. anthracis *chromosomal regions in and around the prophage insertion sites were examined for the presence of putative *attL *and *attR*-like sites. As shown in Figure 2, all prophage sequences are flanked by direct repeats, suggesting probable site-specific recombination mediated insertion of the phage genomes. However, the repeats are of varying lengths for the four phages (lambdaBa04-13 bp, lambdaBa03-108 bp, lambdaBa02-12 bp, and lambdaBa01-65 bp). Also, in lambdaBa0l and lambdaBa03 the direct repeats are not perfect, but have two mismatches (Figure 2). These repeats are present as a single copy at the same chromosomal locus in *B. cereus *group genomes that do not have inserted phages.

**Figure 3:**
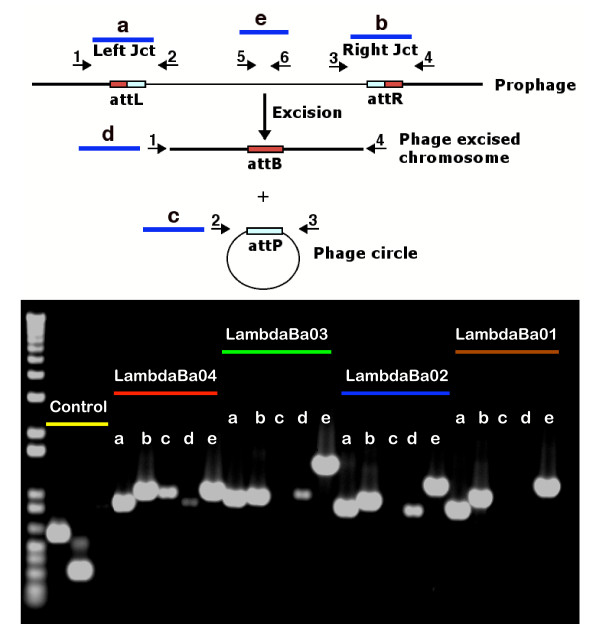
**Top panel: A model for site-specific recombination mediated prophage excision**. The top line shows a schematic representation of the prophage in the chromosome with the *att *sites indicated by colored boxes. Excision of the prophage via site-specific recombination between the *attL *and *attR *sites results in a phage free chromosome (with *attB*) and the phage circle (with *attP*). Small numbered arrows indicate the location of the different primers used to detect prophage excision products and the blue bars (marked a-e) indicate the resulting PCR products. The primers indicated 1–6 correspond to 1-INF, 2-OUTR, 3-OUTF, 4-INR, 5-F and 6-R primers respectively for each prophage. **Bottom panel: Agarose gel electrophoretic analysis of PCR products resulting from prophage excision in *B. anthracis *Sterne strain 34F2**. The set marked as controls corresponds to PCR reactions designed to amplify *pag *gene (pXOl marker), *gmk *gene (chromosomal marker) and a negative control *amp *(pBR322) gene. The four sets of 5 reactions each corresponding to the four prophages is indicated on the lanes by a-e. The corresponding locations of the fragments a-e are shown in panel A.

Lambdoid prophage, upon excision from the chromosome, by a site-specific recombination event between the *attL *and *attR *sites, is expected to generate an extra-chromosomal phage circle and a chromosome with an empty site devoid of the phage genome. In order to test whether *B. anthracis *prophages spontaneously excise from the chromosome, a PCR based assay was designed to detect phage excision products (Figure 3-top panel) [20]. The nucleotide sequences of the primers and the lengths of the expected PCR products are indicated in table 1. Genomic DNA prepared from stationary phase cultures of *B. anthracis *non-pathogenic Sterne strain 34F2 (pXOl^+^, pXO2^-^) was used as template in conjunction with the various primer pairs in PCR reactions and the PCR reaction products were analyzed by agarose gel electrophoresis (Figure 3-bottom panel). PCR products resulting from the prophage-chromosomal junctions and an internal phage fragment (lanes labeled a, b and e) from all 4 prophage regions were visible. However, in three different experiments, the intensities of the PCR products resulting from phage circles (lanes labeled c) and phage free chromosomes (lanes labeled d) of the four phages were weak. In some cases, the PCR products were not visible (lambdaBa03-c, lambdaBa02-c and lambdaBa0lc-d). Similar results were obtained when plasmid preparation or whole cell lysate of strain 34F2 were used as PCR templates (data not shown). These differences could reflect the very low frequencies of the excision events or the inefficient PCR amplification with those primer pairs. In order to address this issue and to verify whether the PCR products seen on the gel result from excision events and not from non-specific amplification, they were cloned into PCR Topo Cloning vector and sequenced. In all cases, [except in lambdaBa02 circle (lane c) and lambdaBa0l phage excised chromosomal site (*attB *site PCR) (lane d)], the sequences of the inserts corresponded to the expected sequence with a single *att *site resulting from the excision of the phage genome from the chromosome (Figure 2). In the case of lambdaBa02 circle and lambdaBa0l phage excised chromosomal site, the concentration of the PCR products was probably too low for successful cloning.

### Excision of the four prophages in stationary phase cultures occurs at low frequencies

**Figure 4:**
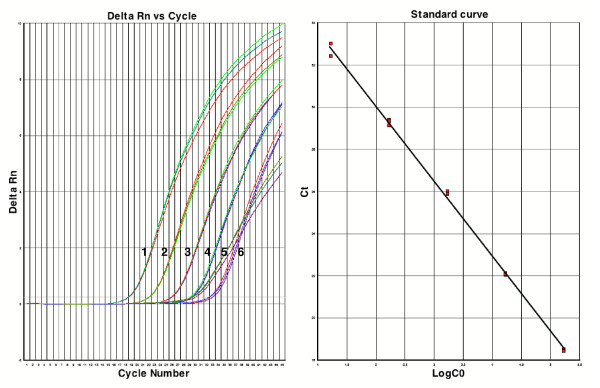
**SYBR green 1 dye PCR assay to determine the frequency of prophage excision**. The amplification profiles in triplicates (marked 1–6) with varying concentrations of the template DNA (34F2 genomic DNA) and primer pairs are shown. The corresponding template concentrations and the fragments amplified are as follows: 1-1 ng/*glp*, 2-100 pg/*glp*, 3–10 pg/*glp*, 4-1 pg/*glp*, 6-100 fg/*glp*, 5-100 ng/Ba03mtF and R. The standard curve generated from the *glp *set is shown on the right panel.

The frequency of spontaneous excision of prophages from *B. anthracis *chromosome was determined using a SYBR-green real-time PCR assay. Genomic DNA extracted from stationary phase cultures of Sterne strain 34F2 was used as template. A chromosomal housekeeping gene (*glp*) was amplified as the control with varying template concentrations to generate a standard curve and the copy number of the PCR products of the phage circles and phage-free empty sites were determined using this standard curve (Figure 4). The phage circles and phage-excised chromosomes were present at very low copies ranging from 8 × 10^-8 ^to 2 × 10^-5 ^per chromosomal copy (*glp*) (Table 2). Cloning and sequencing of the PCR products confirmed their sequences as expected (data not shown). Assuming that the excised phage circle does not undergo replication and that there is one copy of *glp *per cell, the frequencies of excision/cell were estimated to be similar to the copy numbers of the PCR products. Induction of the 34F2 culture with 0.2 μg/ml of mitomycin C (a DNA damaging agent that is known to induce prophages) increased the frequency of excision of lambda Ba04 by about ~250 fold whereas the other three prophages showed no appreciable increase in excision frequencies.

### *B. anthracis *prophages are apparently defective

We conducted a limited analysis to check whether the excised phage genome proceeds to replicate and produce phage particles by testing the unconcentrated culture supernatants for plaque formation on 96 different *Bacillus *sp. strains including the *Bacillus anthracis *Sterne strain 34F2. We hypothesized that any spontaneous repressor mutants of the prophages would be able to overcome the superinfection immunity and form plaques on 34F2. No plaque was visible even with the mitomycin C induced culture supernatant on any of the indicator strains tested. In accordance with this result, unconcentrated culture supernatants did not show any increase in SYBR-green based real time PCR amplicons directed against all four phage specific genes in comparison to a chromosomal gene (*glp*). These amplicons probably resulted from free genomic DNA and not due to the presence of phage particles in culture supernatants since the amount of these amplicons decreased upon DNase treatment of the culture supernatants (data not shown). Electron microscopic analysis of the mitomycin induced and concentrated culture supernatant revealed no intact phage particle or phage components (data not shown).

## Discussion

This study shows that PCR amplicon markers for the four lambdoid prophages found on the *B. anthracis *Ames strain chromosome are present in all of a large number of diverse *B. anthracis *strains tested. Genome sequence analysis could not predict the functionality of the phages since all prophages appear to have a full complement of phage genes. All prophages were demonstrated to be excision proficient and the prophage excision frequency reported here is comparable to that seen in another Gram-positive species, *Lactobacillus lactis *measured by real-time PCR. In that case, the frequency of spontaneous prophage induction varied from 10^-7^-10^-1^/cell depending on the conditions examined [20]. In a limited analysis performed in this study, no viable phage production was observed upon mitomycin C induction as determined by plaque assays using nearly 100 diverse *Bacillus *spp strains as indicators. Although mitomycin C induction exhibited a significant growth inhibition, there was no obvious cell lysis characteristic of phage induction. Direct electron microscopic examination of the concentrated culture supernatants failed to reveal any intact or defective phage particle. This result is in contradiction with an earlier report that polyethylene glycol-concentrated culture supernatants of a *B. anthracis *Sterne strain showed defective phage particles/phage components under electron microscopic examination [21] and is probably due to strain differences or the method used for concentration of the culture supernatant.

There are several possible explanations for the apparent failure in detecting viable phage particles. 1) We have not found an appropriate phage sensitive strain in the panel of 96 strains used in this study for the plaque assay, which might be due to the absence of the phage receptor in these strains. Even if any of the strains possess the receptor, they may lack some other factor needed for phage growth as was shown in the case of γ phage infection of a *B. thuringiensis *strain that possesses the γ phage receptor, the *gamR *gene [22]. Similarly, in another study six phages isolated from *B. anthracis *were screened for their host range and only 2 out of 64 non-*anthracis *strains tested were susceptible to two of the phages [23]. 2) We have not found a repressor mutant that can overcome superinfection immunity and form plaques on 34F2. 3) The fact that all four prophages are simultaneously defective in their ability to produce viable phage particle or lyse the host cell may be indicative of a defective host factor that is involved in phage life cycle.

Although the failure of plaque formation even on closely related species does not necessarily indicate that phages are defective, further work is needed to unequivocally establish this fact. One approach to show that the phages are defective would be to test for plaque formation on the parental carrier strain cured of the respective prophages. Our future studies will focus on testing this possibility.

All four prophages contain genes encoding recombinases and terminal-repeat DNA motifs that may function as attachment (*att*) sites. However, the lambdaBa0l recombinase (GBAA3832) is a pseudogene owing to a frameshift mutation. We hypothesize that lambdaBa0l might recruit some other site-specific recombinase enzyme for its excision, or have a mechanism for frameshift correction during translation. Alternatively, prophage excision might occur via *recA *mediated general recombination pathway, in which case, the frequency of excision will increase with the increase in the length of the repeats and the products will be identical to the site-specific recombination products.

In *Escherichia coli*, prophage excision is classically triggered as a response to DNA damage [24]. In this regard, *B. anthracis *possesses some of key components of an active SOS response system, including a *lexA *ortholog. Despite the fact that there is an intron in the *B. anthracis recA *gene, the encoded protein is still apparently functional [25]. The results of mitomycin C induction experiment described here supports the role of DNA damage in induction of some prophages.

Excision proficient prophage sequences are generally not considered useful targets for bacterial identification because of their instability. However, the constant presence of all four prophages can be advantageous for the definitive discrimination of *B. anthracis *from all its neighbors. There are several schemes for molecular detection of *B. anthracis *DNA using unique markers present on virulence plasmids pXO1 and pXO2 [26-28] but there are relatively few unique chromosomal targets. Targets for confirmation of *B. anthracis *chromosomal DNA include using 16S rDNA sequences [29], *rpoB *[30,31], *gyrB *[6], *gyrA *[32], spore structural protein gene *sspE *[26] and S-layer protein gene *sap *[33] for discrimination of *B. anthracis *from other *Bacillus spp*. Several multiplex PCR assays have also been described for *B. anthracis *detection and discrimination [34-36]. One multiplex PCR assay described earlier entailed amplification of unique fragments identified through suppression subtractive hybridization (SSH) and several of these were located on prophage regions [35]. Because of the close relatedness of the *B. anthracis *to *B. cereus *group strains, many of the PCR based detection techniques are prone to false positive identification of non-*anthracis *strains. The prophages are unique to *B. anthracis *and are present in all *B. anthracis *strains examined so far. Failure to produce the *B. anthracis *specific signals in multiplex PCR would be the result of simultaneous excision of all four prophages, which is highly unlikely. Hence they may offer unique signatures for *B. anthracis *chromosome. From an evolutionary standpoint, it seems possible that the conservation of the phages in all *B. anthracis *may be due to the very recent emergence of the lineage.

## Conclusion

All the *B. anthracis *strains and none of the non-*anthracis *strains tested in this study possess the four prophages, as indicated by the presence of phage specific amplicons suggesting that the prophage regions are unique and can be used for distinguishing *B. anthracis *chromosome from other close relatives. The four prophages excise from the genome at low frequencies; however, in the limited analysis performed in this study, they do not appear to result in production of any viable phage particle or cell lysis, suggesting that the phages may be defective.

## Methods

### Bacterial strains, growth and storage conditions

The bacterial cultures were stored in 15% glycerol at -70°C. The number of strains of each *Bacillus *species from the Biological Defense Research Directorate (BDRD) collection is as follows: 192 *B. anthracis*, five *B. cereus*, two *B. megaterium*, two *B. mycoides*, one *B. pumilus *and one untyped *Bacillus *species. A second collection of strains was obtained from F. Priest (Heriot-Watt University, UK) and contained the following strains: 55 *B. cereus*, three *B. mycoides*, one *B. pseudomycoides*, eight *B. thuringiensis*, two *B. weihenstephanensis *and the following *B. thuringiensis *serovars: three *aizawai*, one *albolactis*, four *canadensis*, one *dakota*, two *darmstadiensis*, one *entomocidus*, one *fluorescens*, two *galleriae*, one *israeliensis*, one *kenyae*, two *kumamotoensis*, one *kurstaki*, three *morrisoni*, one *pakistani*, two *sotto *and one *terminalis*.

### Genomic DNA extraction and PCR

Bacteria were grown in Brain Heart Infusion (BHI) broth and genomic DNA extraction was carried out using Wizard Genomic DNA extraction kit (Promega Corp, Madison, Wi) following the manufacturer's recommended procedures. PCR primers (Table 1) were designed using the eprimer3 software [37] and PCR reactions were carried out using Platinum PCR Supermix (Invitrogen, Inc. Carlsbad, CA) under the following conditions: an initial denaturation cycle at 94°C for 2 min, followed by 32 cycles of denaturation, annealing and extention at 92°C for 30 sec, 56°C for 30 sec, and 72°C for 2 min respectively, followed by a final extension cycle at 72°C for 5 min, in a Dyad Cycler (MJ Research, Inc). The PCR products were initially run on 2% E-gel (Invitrogen, Inc. Carlsbad, CA) and then further verified by running on a 1% agarose gel in TAE buffer.

### Determination of prophage excision

Spontaneous excision of prophages from *B. anthracis *chromosome was determined using genomic DNA prepared from 1.5 ml of overnight culture in BHI medium of a *B. anthracis *Sterne strain, 34F2. The primers and the expected sizes of the PCR products of the phage circles and phage excised chromosomal *attB *sites are listed in Table 1.

### Sequencing of phage excision products

The PCR products of the *in vivo *prophage excision reaction were cloned into Topo cloning vector (Invitrogen, Inc. Carlsbad, CA), and the inserts were sequenced using M13 forward and reverse primers in a CEQ 8000 Genetic Analysis System (Beckman-Coulter, Fullerton, CA) according to the manufacturer's instructions.

### Real-time PCR assay for determination of prophage excision frequencies and the presence of phage particles in culture supernatant

The frequency of excision of the four prophages was determined by a SYBR-green-1 dye real time PCR assay using ABI Prism 7000 Sequence Detection System (Applied Biosystems, Inc. Foster city, CA). The real time PCR primers were designed using the ABI software Primer Express and real-time PCR was performed under the following cycling conditions (10 min at 95°C, 45 cycles of 15 s at 95°C, and 1 min at 60°C). Cycle threshold (CT) values were determined by automated threshold analysis with ABI Prism version 1.0 software. The amplification efficiencies were determined by serial dilution and calculated as *E *= exp^-1/*m*^, where *E *is the amplification efficiency and *m *is the slope of the dilution curve. Under the conditions of the PCR, the efficiency of the PCR reactions with the various primer pairs was comparable, reaching more than 95%. For determination of the presence of free phage particles, unconcentrated culture supernatants were treated with pancreatic DNase I (Sigma-Aldrich) at 1 mg/ml for 1–3.5 hrs at 37°C and used as template in real time PCR assays using individual primers targeting each prophage.

### Electron microscopy of culture supernatants

*B. anthracis *Sterne strain, 34F2, was grown in BHI to an OD600 of ~0.2 to 0.4 and induced with mitomycin C (0.5 μg/ml) for 16 hrs. The culture was spun at 8000 rpm for 10 min, the supernatant filtered through 0.45 μm syringe filter (Millex-HV PVDF-Millipore Corp, Medford, MA) and further concentrated 15 × using Amicon Centriplus YM10 (MWCO 10 KDa) filtration units (Millipore Corp, Medford, MA). The concentrated lysate was used for electron microscopic examination. Ten μ1 of each sample was adsorbed onto formvar coated 300-mesh copper grid for 1 minute and excess fluid was wicked away with filter paper. Grids were then washed on 3 droplets of distilled water and stained for 1 minute with 1% of phosphotungstic acid or 1% of uranyl acetate. Grids were examined in a Jeol JEM-1200EX II transmission electron microscope operated at 80 kV. Each grid was scanned for 20 minutes covering more than 70% of the total viewing fields.

## Authors' contributions

SS contributed in designing and carrying out the experiments, MDC, FDM carried out strain collection, PCR analysis, DBF and AA contributed in the bioinformatics analysis, DRG, AM, LWB contributed ideas for the project and the manuscript was written by SS and TDR.

## Figures and Tables

**Table 1:** Primers Used in PCR screening of Phage Genes and Phage Excision Products

			Primer	PCR product	Location
Primer Name	ORF/gene product	Sequence 5' to 3'	Length	Size (bp)	

ORF01192F^a^	BA0479-hypothetical	AAACCCTGGGACCTCTGAAC	20	1002	prophage 04
ORF01192R^a^	BA0479-hypothetical	GGAAGAATCGCACGACCATC	20		prophage 04
ORF02190F^a^	BA5356-Terminase, large subunit	GACATCGTTGCACCTTCACAAG	22	1462	prophage 03
ORF02190R^a^	BA5356-Terminase, large subunit	CCAAATGTCGAGCATCTTGTTC	22		prophage 03
ORF03655 F^b^	BA4094-Terminase, large subunit	TTGATCGATCCATCTCCTGAAC	22	1192	prophage 02
ORF03655 R^b^	BA4094-Terminase, large subunit	GATCAACTTTAGCCTGCGGAAC	22		prophage 02
Ba02 #7 For^c^	BA4094-Terminase, large subunit	CAAAGACAGATCCACCTGGAC	21	304	prophage 02
Ba02#7 Rev^c^	BA4094-Terminase, large subunit	CAAAGGGAGGTTCAGCATCTC	21		prophage 02
ORF03991 F^a^	BA3805-Acyl transferase	AGTTGCATGCCCAGTTCTTG	20	1205	prophage 01
ORF03991 R^a^	BA3805-Acyl transferase	CTGCGTGACTGGAATCCCTTAC	22		prophage 01
Ba04INF	LambdaBa04 left junction	CCAGTTGAATCCAGAACAAACG	22	871	prophage 04
Ba04OUTR	LambdaBa04 left junction	GGGCAGTCATACGAGGATAATG	22	(961,865)^d^	prophage 04
Ba04OUTF	LambdaBa04 right junction	CCTTCGCTTTGAATTCCTTCTC	22	997	prophage 04
Ba04INR	LambdaBa04 left junction	GAATTGTAACGAGCATGGAAGC	22		prophage 04
Ba03INF	LambdaBa03 left junction	ACGTTACCCCTATTTCCGAAGC	22	938	prophage 03
Ba03OUTR	LambdaBa03 left junction	CATTTTAATGCGCCCACGAC	20	(923, 952)^d^	prophage 03
Ba03OUTF	LambdaBa03 right junction	TTCCACATCTTCCTTCAGCAAC	22	977	prophage 03
Ba03INR	LambdaBa03 left junction	GGCCGTACTGGCTTAACTTCTG	22		prophage 03
Ba02INF	LambdaBa02 left junction	TCACTTGCCAGTCTTGACCTTG	22	891	prophage 02
Ba02OUTR	LambdaBa02 left junction	GGTGCATAAGGCGGTAAAGATG	22	(861,962)^d^	prophage 02
Ba02OUTF	LambdaBa02 right junction	GGCGAGGTATTAGCTTTACAGTGG	24	976	prophage 02
Ba02INR	LambdaBa02 left junction	GTCCATCTTCACTGCCGAAAC	21		prophage 02
Ba011NF	LambdaBa01 left junction	ACAAATTCAGTTGCGCTTCC	20	1053	prophage 01
Ba010UTR	LambdaBa01 left junction	TGCAGCACCTACACTGAAACAAG	23	(878, 1047)^d^	prophage 01
Ba010UTF	LambdaBa01 right junction	CGATGGAAAGTTCTTACCGAAG	22	915	prophage 01
Ba011NR	LambdaBa01 left junction	ATGATGCTCGTCACTTCATCG	21		prophage 01
gmk F	BC guanylate kinase, putative	ATTTAAGTGAGGAAGGGTAGG	21	500	chr
gmk R	BC guanylate kinase, putative	GCAATGTTCACCAACCACAA	20		chr
PA1 For	Protective antigen	ATCACCAGAGGCAAGACACC	20	311	pXO1
PA1 Rev	Protective antigen	CCATTGTTTCAGCCCAAGTT	20		pXO1
bla F	Amp resistance	TTACCAATGCTTAATCAGTGAGGC	24	861	plasmid
bla R	Amp resistance	ATGAGTATTCAACATTTCCGTGTC	24		plasmid
Primers Used in Real Time PCR for determination of Phage Excision Frequencies					
Bce_glpR	BC glycerol kinase	GCAGTAGCGGTTGCAGCATA	20	150	chr
Bce_glpF	BC glycerol kinase	CCCGATAATTGCCCCAATC	19		chr
Ba04circF	LambdaBa04 circle junction	TCAAACCCATCAACAATTTCATTG	24	129	Extra-chr
Ba04circR	LambdaBa04circle junction	CAATAGAATGCACAACAGTGACATAAGT	28		Extra-chr
Ba03circF	LambdaBa03 circle junction	CAAGGGTTGTAGCTGATAGCTCATT	25	170	Extra-chr
Ba03circR	LambdaBa03 circle junction	TGACAAAGTTCAGTCGATTTTTTTCT	26		Extra-chr
Ba02circR	LambdaBa02 circle junction	GACCACAACTTGTACCACATTTATTATTT	29	166	Extra-chr
Ba02circF	LambdaBa02 circle junction	CCGCAATATAGGTGGTATAATGCA	24		Extra-chr
Ba01circF	LambdaBa01 circle junction	CCCACAAAATAAAAAAACCCTCAA	24	150	Extra-chr
Ba01circR	LambdaBa01 circle junction	ACGTTTTTGGCGCAATTTAAA	21		Extra-chr
Ba04mtF	LambdaBa04 deleted junction	AGCACGTGATGTACAAGCGTTAA	23	150	φ free chr
Ba04mtR	LambdaBa04 deleted junction	TATTCCCTCATATCATGAGGGAATATG	27		φ free chr
Ba03mtF	LambdaBa03 deleted junction	TGGCCAAATCAAAACTGGTTCT	22	165	φ free chr
Ba03mtR	LambdaBa03 deleted junction	TTCTCACGGTCTGTCGTTATTTTC	24		φ free chr
Ba02mtF	LambdaBa02 deleted junction	ATATCACCTCAAGGCAACAAACAA	24	150	φ free chr
Ba02mtR	LambdaBa02 deleted junction	GGTAATCTCTCCTTTCGATGTAGCA	25		φ free chr
Ba01mtF	LambdaBa01 deleted junction	CATTAGGAGATCACTTACTTGAGCACTT	28	161	φ free chr
Ba01mtR	LambdaBa01 deleted junction	CACAAATAAAAAAACCTTGATACCGTAGT	29		φ free chr
Primers Used in Real time PCR screening of Prophage Genes			Primer	PCR product	Location
Ba04-RT-F	BA0479-hypothetical	TATAATGGGCACTCCATTTTGGT	23	150	prophage 04
Ba04-RT-R	BA0479-hypothetical	TCCACAGTGGCATTTACCTTTG	22		prophage 04
Ba03-RT-F	BA5356-Terminase, large subunit	TCCTATCGAGAATGGGTTCAACTAT	25	150	prophage 03
Ba03-RT-R	BA5356-Terminase, large subunit	GCGTCCCTACCGTTCAACTG	20		prophage 03
Ba02-RT-F	BA4094-Terminase, large subunit	TGCGTTTTATCATGGAAAATGC	22	150	prophage 02
Ba02-RT-R	BA4094-Terminase, large subunit	TGAGCCAGGTGCTCGTGTT	19		prophage 02
Ba01-RT-F	BA3805-Acyl transferase	CACTTGAATCAACTGGTATCGTGAA	25	150	prophage 01
Ba01-RT-R	BA3805-Acyl transferase	AAATCCAAATTGAGGCATATGATGA	25		prophage 01

^a^simplex and multiplex; ^b^simplex only; ^c^multiplex only; ^d^sizes of PCR products of phage circle and phage excised chromosomal junction respectively

**Table 2:** Frequency of excision of the four prophages from *B. anthracis *chromosome

Excision product	Frequency of excision*
LambdaBa04 circle	2.1 × 10^-5^
LambdaBa04 empty site	ND
LambdaBa03 circle	6.4 × 10^-6^
LambdaBa03 empty site	3.7 × lO^-6^
LambdaBa02 circle	7.7 × 10^-8^
LambdaBa02 empty site	2.8 × 10^-7^
LambdaBa01 circle	2.2 × 10^-6^
LambdaBa01 empty site	3.4 × 10^-7^

ND-Not determined; *glp std-standard curve for estimating the copy number of different PCR products was generated using *glp *amplicon (representative of a single copy of chromosome)
